# miR-145 inhibits tumor growth and metastasis by targeting metadherin in high-grade serous ovarian carcinoma

**DOI:** 10.18632/oncotarget.2522

**Published:** 2014-10-23

**Authors:** Ruifen Dong, Xiaolin Liu, Qing Zhang, Zhijun Jiang, Yingwei Li, Yuyan Wei, Yinuo Li, Qifeng Yang, Jinsong Liu, Jian-Jun Wei, Changshun Shao, Zhaojian Liu, Beihua Kong

**Affiliations:** ^1^ Department of Obstetrics and Gynecology, Qilu Hospital, Shandong University, Jinan, Shandong, China; ^2^ Department of Cell Biology, Shandong University School of Medicine, Jinan, Shandong, China; ^3^ Ministry of Education Key Laboratory of Experimental Teratology and Department of Molecular Medicine and Genetics, Shandong University School of Medicine, Jinan, China; ^4^ Department of Breast Surgery, Qilu Hospital, Shandong University, Jinan, Shandong, China; ^5^ Department of Pathology, Northwestern University School of Medicine, Chicago, IL, USA; ^6^ Department of Pathology, The University of Texas MD Anderson Cancer Center, Houston, TX, USA

**Keywords:** miR-145, MTDH, p53, HGSOC, metastasis

## Abstract

High-grade serous ovarian carcinoma (HGSOC), the most common and aggressive subtype of epithelial ovarian cancer, is characterized by TP53 mutations and genetic instability. Using miRNA profiling analysis, we found that miR-145, a p53 regulated miRNA, was frequently down-regulated in HGSOC. miR-145 down-regulation was further validated in a large cohort of HGSOCs by qPCR. Overexpression of miR-145 in ovarian cancer cells significantly suppressed proliferation, migration and invasion in *vitro* and inhibited tumor growth and metastasis in *vivo*. Metadherin (MTDH) was subsequently identified as a direct target of miR-145, and was found to be significantly up-regulated in HGSOC. Furthermore, overexpression of MTDH rescued the inhibitory effects of miR-145 in ovarian cancer cells. Finally, we found that high level of MTDH expression correlated with poor prognosis of HGSOC. Therefore, lack of suppression of MTDH by miR-145 when p53 is dysfunctional leads to increased tumor growth and metastasis of HGSOC. Our study established a new link between p53, miR-145 and MTDH in the regulation of tumor growth and metastasis in HGSOC.

## INTRODUCTION

Epithelial ovarian cancer is the most lethal gynecologic malignancy with several histological subtypes. High-grade serous ovarian carcinoma (HGSOC or HGSC), the most common and aggressive subtype, has the highest mortality rate, with a 5-year survival rate of only 30%[1]. The advance stage at the time of diagnosis and relapse due to chemoresistance are the major reasons for poor prognosis[1]. HGSOC is characterized by TP53 mutations and genetic instability[2]. According to The Cancer Genome Atlas (TCGA), TP53 and BRCA1/2 mutations are the major genetic alterations occuring in 96% and 22% of HGSOC cases, respectively [3]. A genetically defined mouse model which closely mimic to human HGSOC was established recently by inactivating BRCA1, p53 and PTEN genes[4]. Despite those encouraging developments, the pathogenesis of HGSOC remains poorly understood and no biomarkers for prediction of response to therapy are yet available in clinical.

miRNAs are found to play important roles in tumorigenesis and metastasis in human cancers[5, 6]. Recent reports showed that miRNAs were aberrantly expressed in HGSOC[7]. We previously showed that miR-182 was overexpressed in HGSOC and miR-182 overexpression conferred a potent oncogenic property by targeting BRCA1 and MTSS1[8]. Using miRNA expression profiling in HGSOC, we found that a p53 regulated miRNA, miR-145, was down-regulated in most HGSOC cases. miR-145 expression level has been shown to be decreased in various human cancers such as breast cancer [9] and ovarian cancer[7]. miR-145 was reported to inhibit tumor angiogenesis and metastasis in various cancers by targeting several protein coding genes such as c-myc and N-cadherin[10–12]. However, the patho-biological significance of aberrant miR-145 expression in HGSOC has not been fully elucidated.

In the current study, we investigated the functional role of miR-145 in HGSOC, both in *vitro* and in *vivo*. We found that overexpression of miR-145 in ovarian cancer cells significantly suppressed proliferation, migration and invasion in *vitro* and inhibited tumor growth and metastasis in *vivo*. Furthermore, metadherin (MTDH), an oncogene that was highly expressed in breast cancer[13] and ovarian cancer[14], was identified as a direct target of miR-145.

## RESULTS

### Down-regulation of miR-145 in HGSOC tissues and ovarian cancer cell lines

**Figure 1:**
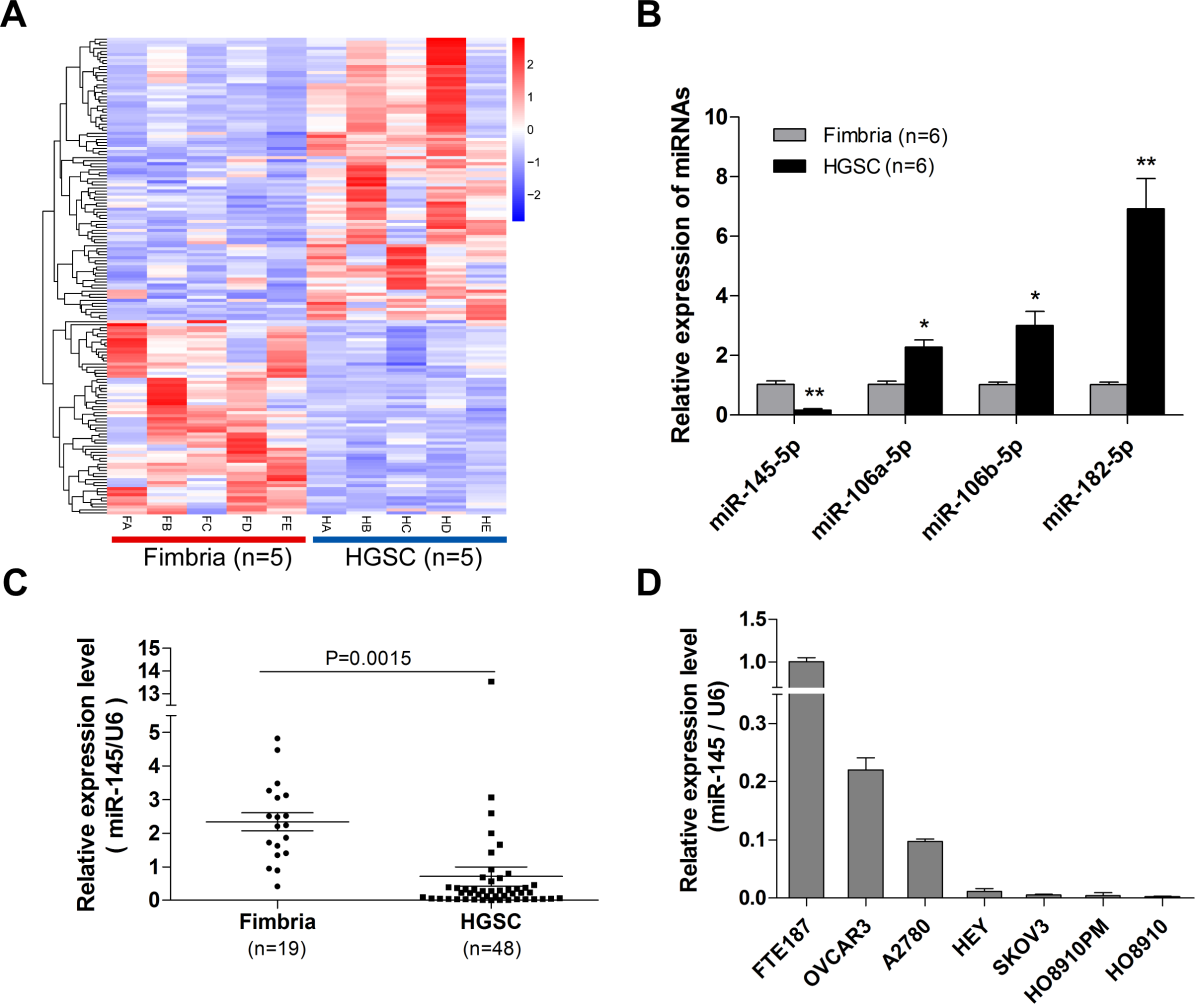
Down-regulation of miR-145 in ovarian cancer tissues and cell lines **(A)** Heat map illustrates the dysregulated miRNAs in HGSOC compared with normal fimbria according to the five pairs of tissue microarray results. HA-HE represent the 5 high grade serous cancer samples, and FA-FE represent the 5 normal fimbria samples. **(B)** Validation of four microRNAs differential expression in normal fimbria and HGSOC with qPCR assay. (C) QPCR validation of miR-145 expression in fresh fimbria and HGSOC in a large cohort. **(D)** Relative miR-145 expression levels of six ovarian cancer cell lines and one normal fallopian tube epithelial cell line were examined with qPCR assay. *P<0.05, **P <0.01.

The Cancer Genome Atlas (TCGA) project showed that TP53 mutations were detected in almost all HGSOC (96%)[3]. Since p53 regulates the expression of many miRNAs[15], we speculated that p53 mutations may contribute to the tumorigenesis and metastasis of HGSOC through p53 regulated miRNAs. To identify aberrantly expressed miRNAs in HGSOC, we conducted a global miRNA expression analysis in HGSOC and normal fallopian tube fimbria (five cases each, Figure 1A, Table S1). We found that one of p53 regulated miRNAs, miR-145, was down-regulated in HGSOC. This finding revealed by microarray profiling was confirmed with qPCR (Figure 1B). Three significantly up-regulated miRNAs identified in our previous study were also examined, indicating the reliability of current profile data. To determine whether miR-145 was down-regulated in most HGSOC, we examined miR-145 expression level in a larger sample. As shown in Figure 1C, miR-145 was significantly down-regulated in HGSOC (n=48) compared with fimbria (n=19). We further measured miR-145 expression in six ovarian cancer cell lines and one immortalized fallopian tube epithelial cell line. We found that the expression levels of miR-145 in ovarian cancer cell lines were much lower than in normal cell line (Figure 1D). These results indicate that miR-145 is down-regulated in both HGSOC tissues and cancer cell lines.

### Overexpression of miR-145 suppresses proliferation of ovarian cancer cells in *vitro*

**Figure 2:**
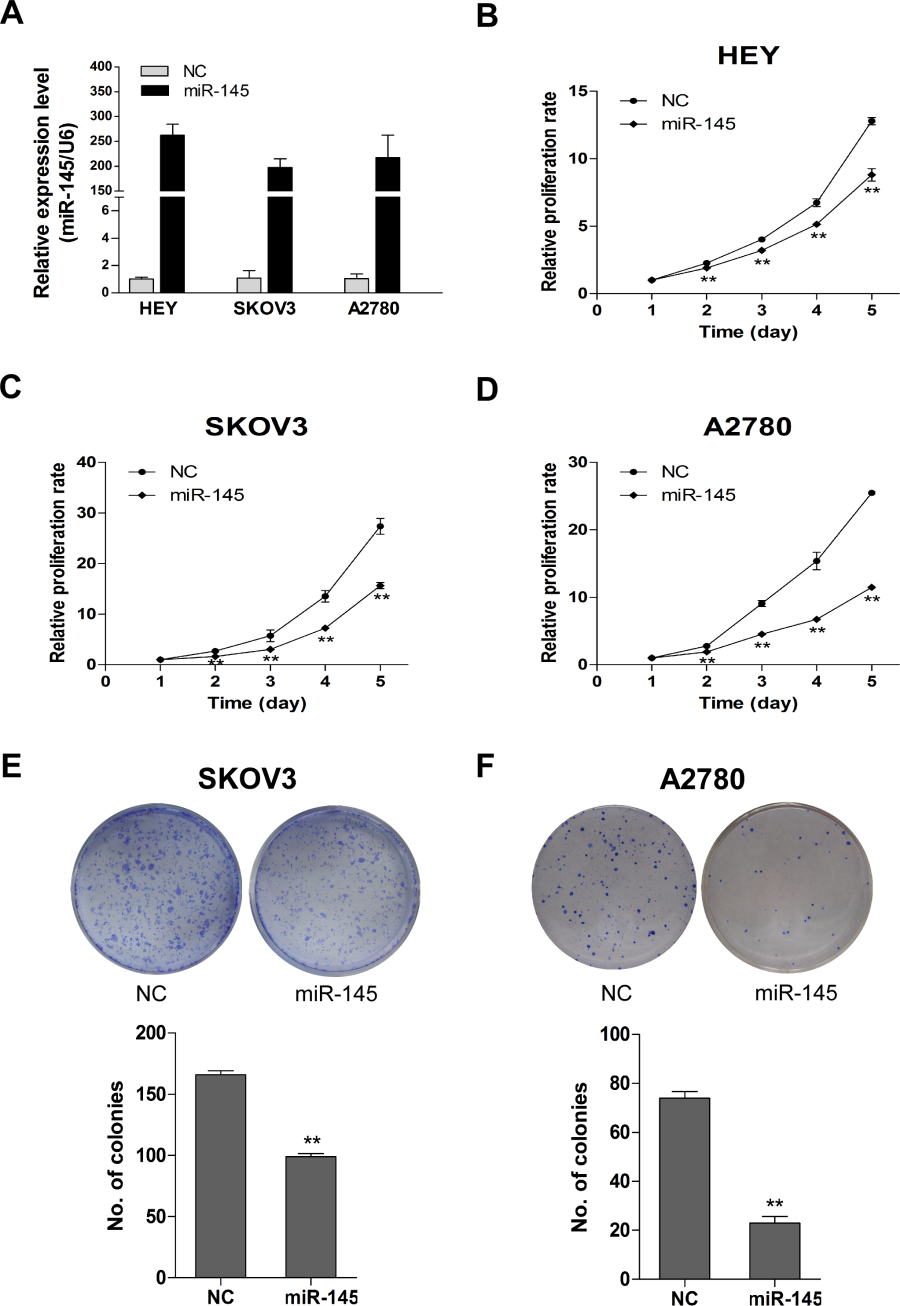
Stable miR-145 overexpression suppresses cell proliferation and clonogenic ability in *vitro* **(A)** The overexpression of miR-145 in HEY, SKOV3 and A2780 cells was validated with qPCR. **(B-D)** Effects of ectopic expression of miR-145 on the proliferation of HEY, SKOV3 and A2780 cells were examined with MTT assay and shown as growth curve. **(E-F)** Effects of overexpression of miR-145 on the clonogenic ability of SKOV3 and A2780 cells. The upper panel represents dishes by colony formation assay, and the lower panel illustrates the number of colonies formed. Columns: average of three independent experiments; bars: se. **P<0.01.

To investigate the possible role of miR-145 in ovarian cancer, we established three ovarian cancer cell lines with stable miR-145 overexpression (Figure 2A). We then measured the inhibitory effect of miR-145 on ovarian cancer cell proliferation. As shown in Figure 2B-D, overexpression of miR-145 significantly suppressed the proliferation of ovarian cancer cells as measured by MTT assay. The inhibitory effect of miR-145 on ovarian cancer cell proliferation was further evaluated by clonogenic assay. As shown in Figure 2E-F, the colony-forming efficiency was significantly reduced in miR-145 overexpressed cells than in control cells. These findings suggest that miR-145 suppresses proliferation of ovarian cancer cells in *vitro*.

### miR-145 suppresses migration and invasion of ovarian cancer cells in *vitro*

**Figure 3:**
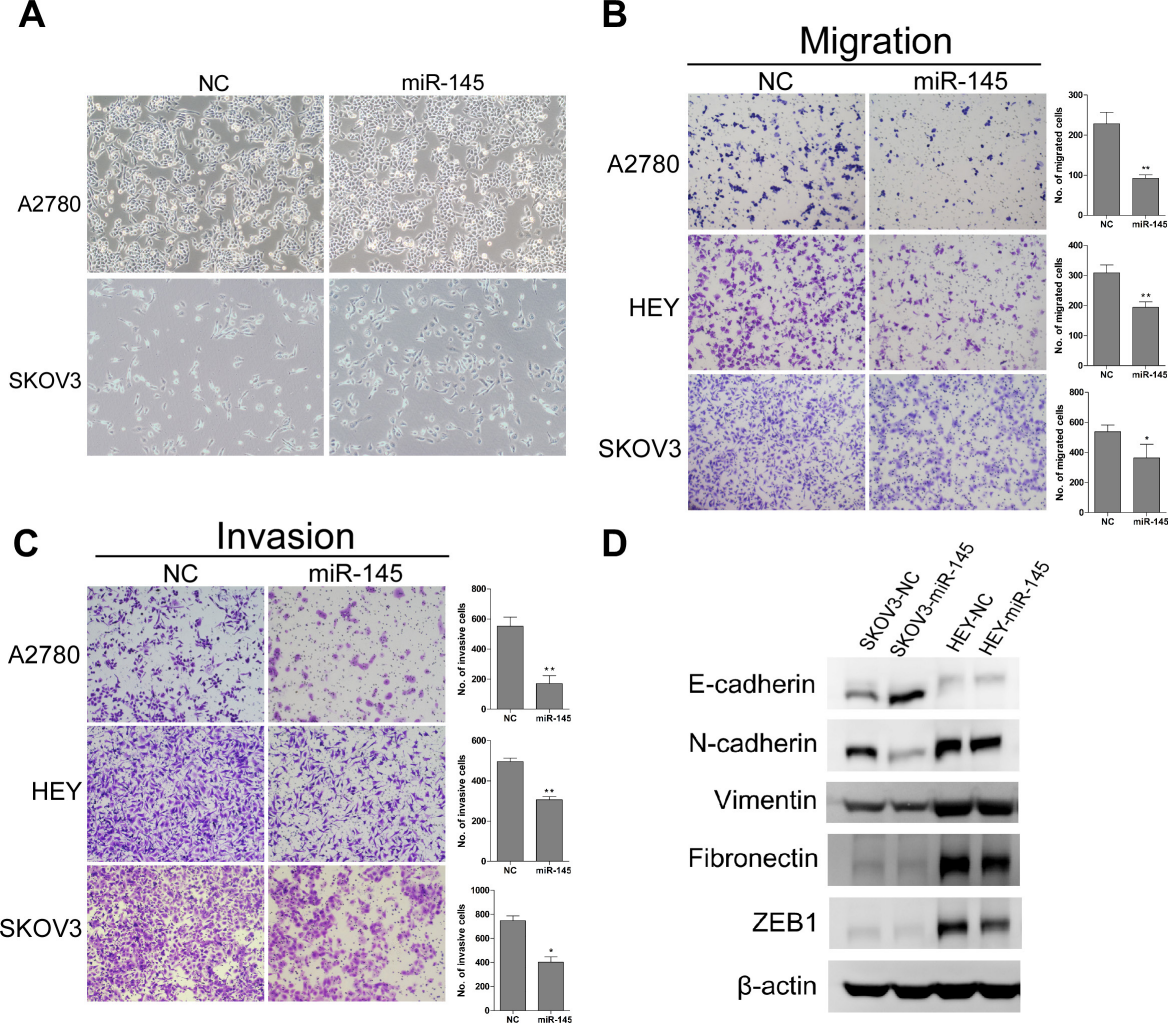
Ectopic expression of miR-145 suppresses ovarian cancer cell migration and invasion in *vitro* and induced mesenchymal to epithelial transformation(MET) **(A)** Morphological changes in miR-145 overexpressed cells compared to control cells. **(B)** Effects of ectopic expression of miR-145 on the migration of A2780, HEY and SKOV3 cells. **(C)** Effects of ectopic expression of miR-145 on the invasion of A2780, HEY and SKOV3 cells. **(D)** MET related markers show different expression in SKOV3 and HEY cells with and without miR-145 overexpression. bars: se. *P*<0.05, **P<0.01.

To test whether miR-145 overexpression suppresses tumor cell migration and invasion, we first examined the morphological changes in miR-145 overexpressed cells. As shown in Figure 3A, A2780 and SKOV3 cells overexpressing miR-145 exhibited epithelial morphology. The migration and invasion were then measured by transwell assay. We found that miR-145 could significantly suppress migration (Figure 3B) and invasion (Figure 3C) in all three ovarian cancer cells lines. To further investigate whether the inhibitory effect of miR-145 on migration and invasion was mediated by mesenchyme to epithelia transition (MET), we examined the expression of several MET markers. As expected, miR-145 overexpression increased the expression level of epithelial marker (E-cadherin) and decreased the levels of mesenchymal markers (Figure 3D). Taken together, these findings suggest that miR-145 could impede invasion mediated by MET in *vitro*.

### miR-145 inhibits tumor growth and metastasis of xenografts

**Figure 4:**
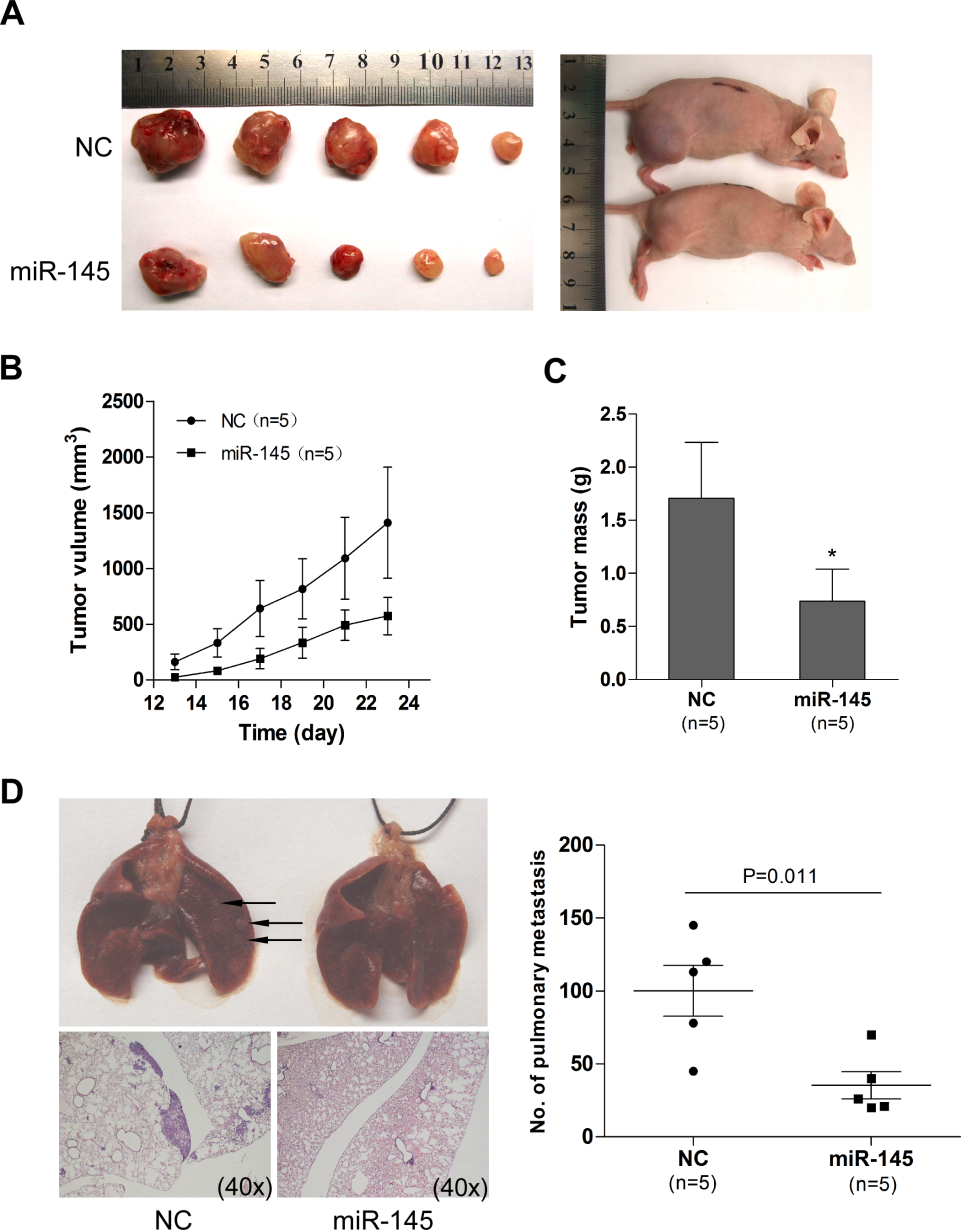
miR-145 overexpression inhibits tumor growth and metastasis in a xenograft model **(A)** Photographs illustrate representative tumors in xenografts of A2780 cell lines with miR-145 overexpression (miR-145) compared to tumors without miR-145 overexpression (NC). **(B)** Growth curves for tumor volumes in xenografts of nude mice with A2780 cell line with and without miR-145 overexpression (each consists of 5 mice), measured from day 13 to day 23. **(C)** miR-145 overexpression results in a decline of tumor weight. **(D)** Representative images of lungs (arrows illustrates the visible nodules)and hematoxylin and eosin (HE)-staining of lungs isolated from mice that received tail vein injection of SKOV3 cell line with and without miR-145 overexpression. Each group contains 5 mice. Plot graph illustrates the numbers of pulmonary metastatic nodules under microscope were counted and analyzed with Student's t-test. All data are shown as mean±se. *P<0.05.

We next examined whether miR-145 overexpression could suppress ovarian cancer tumor growth and metastasis in *vivo*, in nude mice. A2780 ovarian cancer cells with and without miR-145 overexpression were subcutaneously inoculated in nude mice (n=5 for each group). We found that the tumor sizes derived from A2780-miR-145 overexpressing cells were smaller than those in control group A2780-NC (Figure 4A and 4B). Additionally, the tumors formed from the A2780-miR-145 cells weighed significantly less when compared to A2780-NC (Figure 4C). To examine tumor metastasis, we injected, via tail vein, SKOV3 cells with and without miR-145 overexpression into nude mice. After 6 weeks mice were anesthetized and mouse lungs were harvested, fixed and dissected. Four out of five mice transplanted with SKOV3-NC cells had visible pulmonary metastatic nodules, while no mouse in the SKOV3-miR-145 group showed visible lung metastasis (Figure 4D). These results suggest that overexpression of miR-145 inhibits tumor growth and metastasis in *vivo*.

### MTDH is a direct target gene of miR-145

**Figure 5:**
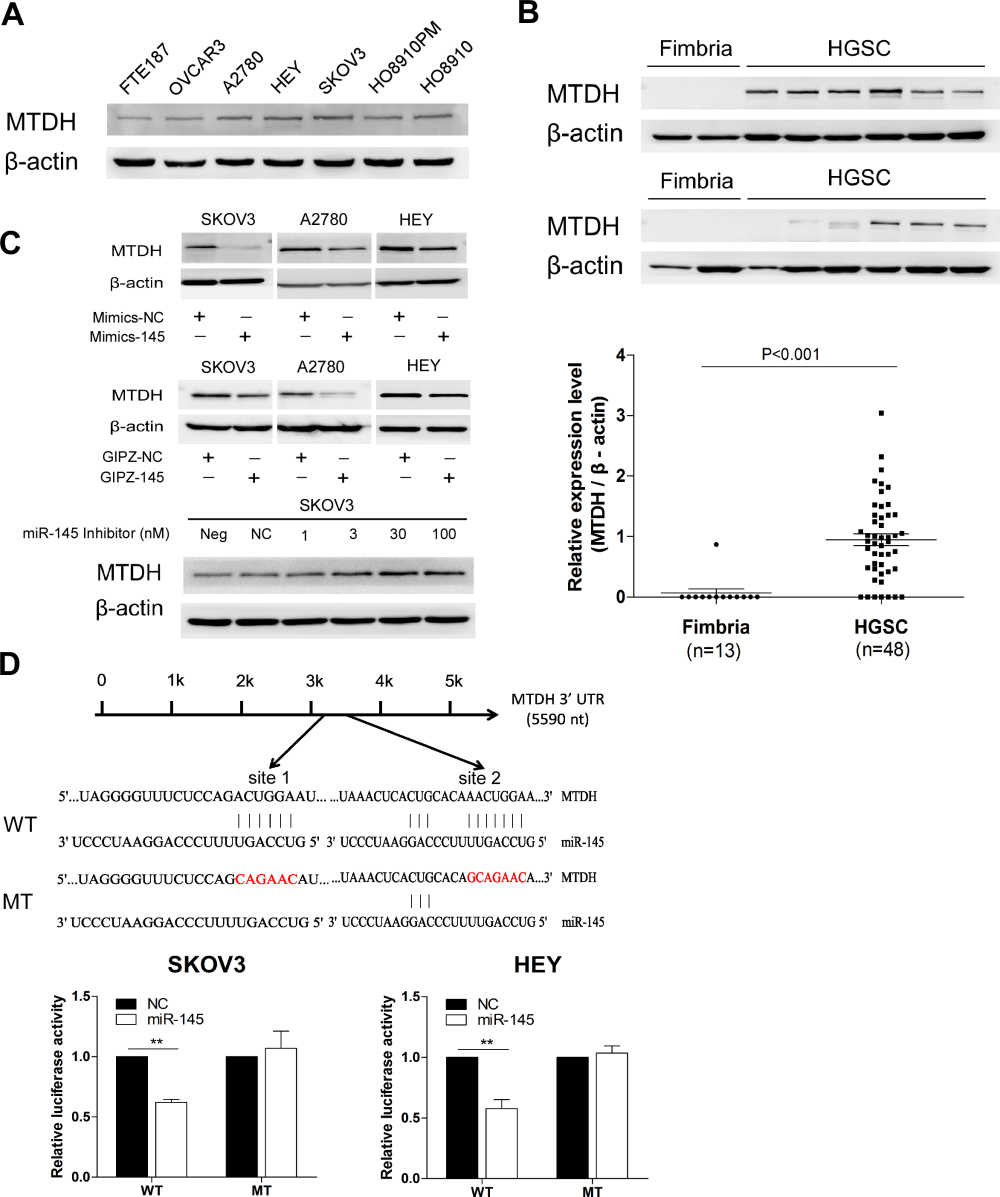
Oncogene MTDH is specifically targeted by miR-145 **(A)** Western blot analysis of MTDH expression in ovarian cancer cell lines and in normal fallopian tube cell FTE187. **(B)** Western blot analysis of MTDH in HGSOC tissue samples (n=48) compared to normal fimbria, and the plot graph reveals the statistical result of expression of MTDH in all samples normalized and quantified (mean±se). **(C)** Western blot analysis reveals an inverse correlation of MTDH expression with miR-145 expression in malignant cell lines. Upper panel: transient transfection of miR-145 mimics into cells reduced MTDH protein expression in SKOV3, A2780 and HEY cell lines. Middle panel illustrates that stable overexpression of miR-145 also induced MTDH down-regulation in three cell lines. Lower panel: different concentration of miR-145 inhibitor transfection gradually caused increased expression of MTDH in SKOV3 cell when compared to cell with no transfection (neg) or transfection with inhibitor negative control (NC). **(D)** Luciferase reporter assay revealed miR-145 suppressed MTDH 3′UTR luciferase activity. The skematic graph shows two predicted binding sites of miR-145 in MTDH 3′UTR and their sequences. Histobars illustrate the relative luciferase activity in SKOV3 and HEY cells with and without miR-145 overexpression when transfected with WT and MT luciferase plasmids (mean±se). **p<0.01.

To identify the functional target of miR-145 in HGSOC, we analyzed proteins that were up-regulated in HGSOC and the predicted targets of miR-145 from publicly available databases as PicTar, TargetScan and miRDB using Venn diagram (data not shown). MTDH was among the predicted target genes of miR-145. MTDH was reported to be overexpressed in breast cancer and to be associated with poor prognosis, due to its participation in tumorigenesis, metastasis and chemoresistance[13, 16]. To investigate whether MTDH contributes to HGSOC, we first examined the MTDH expression in ovarian cancer cell lines and primary HGSOC tissues. We found that MTDH level was significantly higher in ovarian cancer cells than in normal epithelial cells (Figure 5A). Consistently, MTDH showed much higher level of protein expression in primary HGSOC tissues(n=48) compared to normal fimbria (n=13) by western bolt (Figure 5B). We next tested whether MTDH could be directly targeted by miR-145. There are two possible binding sites for miR-145 in the 3′UTR of MTDH. To test whether MTDH expression was regulated by miR-145, we compared MTDH expression in cells with and without miR-145 overexpression. As shown in Figure 5C, both transient and stable ectopic overexpression of miR-145 led to the inhibition of MTDH expression. In contrast, miR-145 inhibitor dose-dependently restored MTDH expression. To confirm that MTDH is a direct target of miR-145, we then introduced MTDH 3′UTR and corresponding mutant counterparts into pmiRGLO vector. These reporter vectors were then transfected into cells with and without miR-145 overexpression. We found that miR-145 overexpression reduced the luciferase activity in cells transfected with the wild type 3′UTR of MTDH but not in cells with mutant 3′UTR in two cell lines (Figure 5D). Taken together, these results indicate that MTDH is a direct downstream target of miR-145 and MTDH overexpression in HGSOC may be attributed to the reduced expression of miR-145.

### Overexpression of MTDH rescues the inhibitory effects of miR-145 on ovarian cancer cells

**Figure 6:**
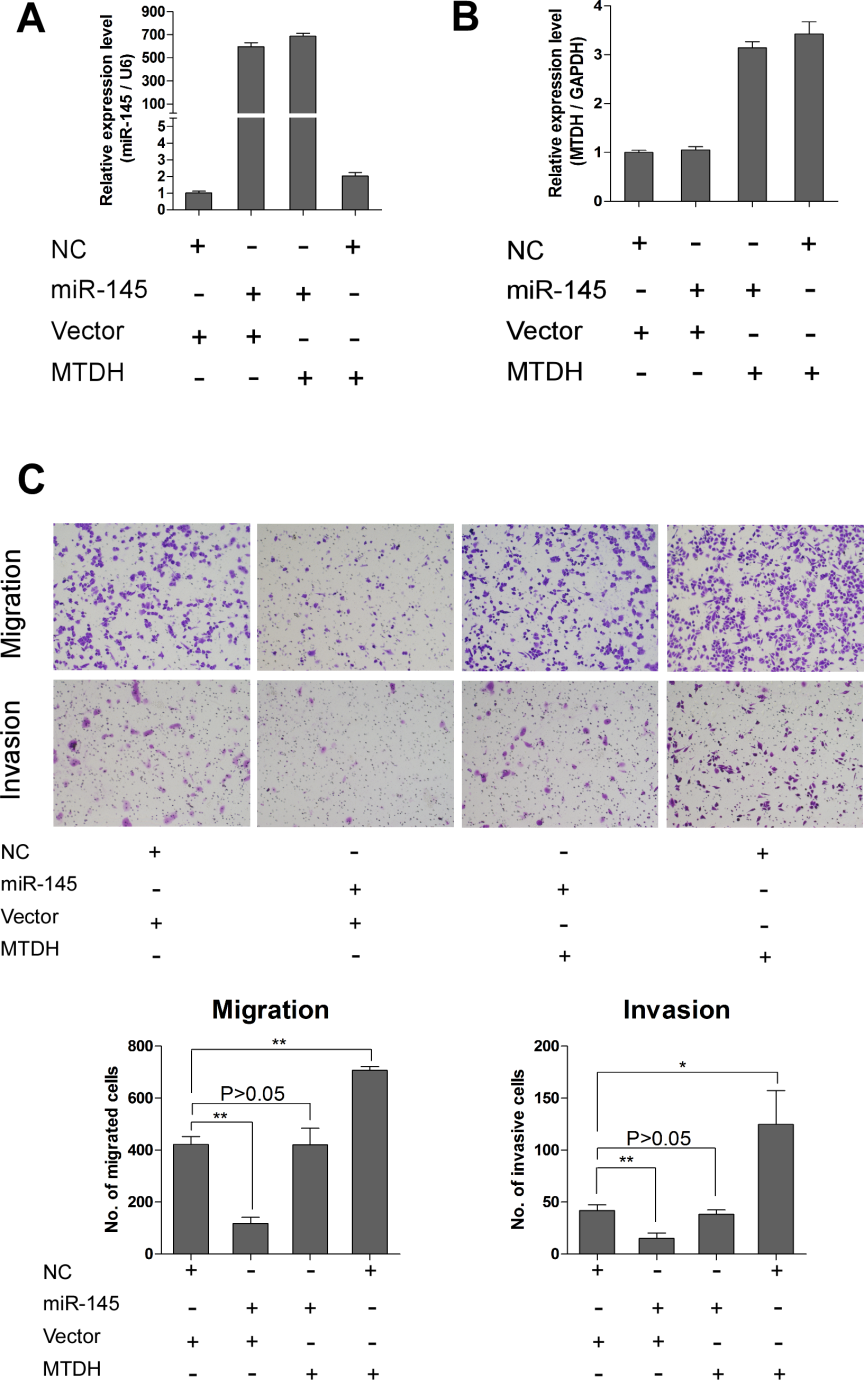
Overexpression of MTDH rescues the inhibitory effects of miR-145 on ovarian cancer cells **(A)** QPCR analysis shows expression of miR-145 in cells transfected with different vectors. **(B)** QPCR analysis shows expression of MTDH in cells transfected with different vectors. MTDH means pBABE-hygro-MTDH overexpression plasmid, and vector is for pBABE-hygro-vector. **(C)** Transwell assay reveals that reduction of migration and invasion caused by expression of miR-145 can be rescued by introduction of MTDH. *P<0.05, **P<0.01.

To explore whether MTDH is involved in miR-145 induced suppression of ovarian cancer cell proliferation and invasion, we performed rescue experiment of MTDH overexpression in miR-145 ectopically expressed cells and corresponding control cells. The MTDH overexpression construct was designed as containing only coding sequences of MTDH without 3′UTR. We used this MTDH overexpressing construct and corresponding vector to transfect A2780 cells with stably overexpressing miR-145 or negative control. We established four stable subgroups of cells and measured the expression level of miR-145 and MTDH by qPCR. As shown in Figure 6A and 6B, we successfully established these four subgroups of cells for rescue experiment. We then attempted to test whether restoration of MTDH could reverse the miR-145 mediated inhibition of metastatic ability of ovarian cancer cells. As can been seen from Figure 6C, overexpression of MTDH with a cDNA without 3′UTR could partially abrogated miR-145 mediated suppression on migration and invasion of ovarian cancer cells. These observations support that MTDH is one of the major targets mediating the miR-145 effects on tumor metastasis of human ovarian cancer.

### p53 represses MTDH through induction of miR-145 in HGSOC

**Figure 7:**
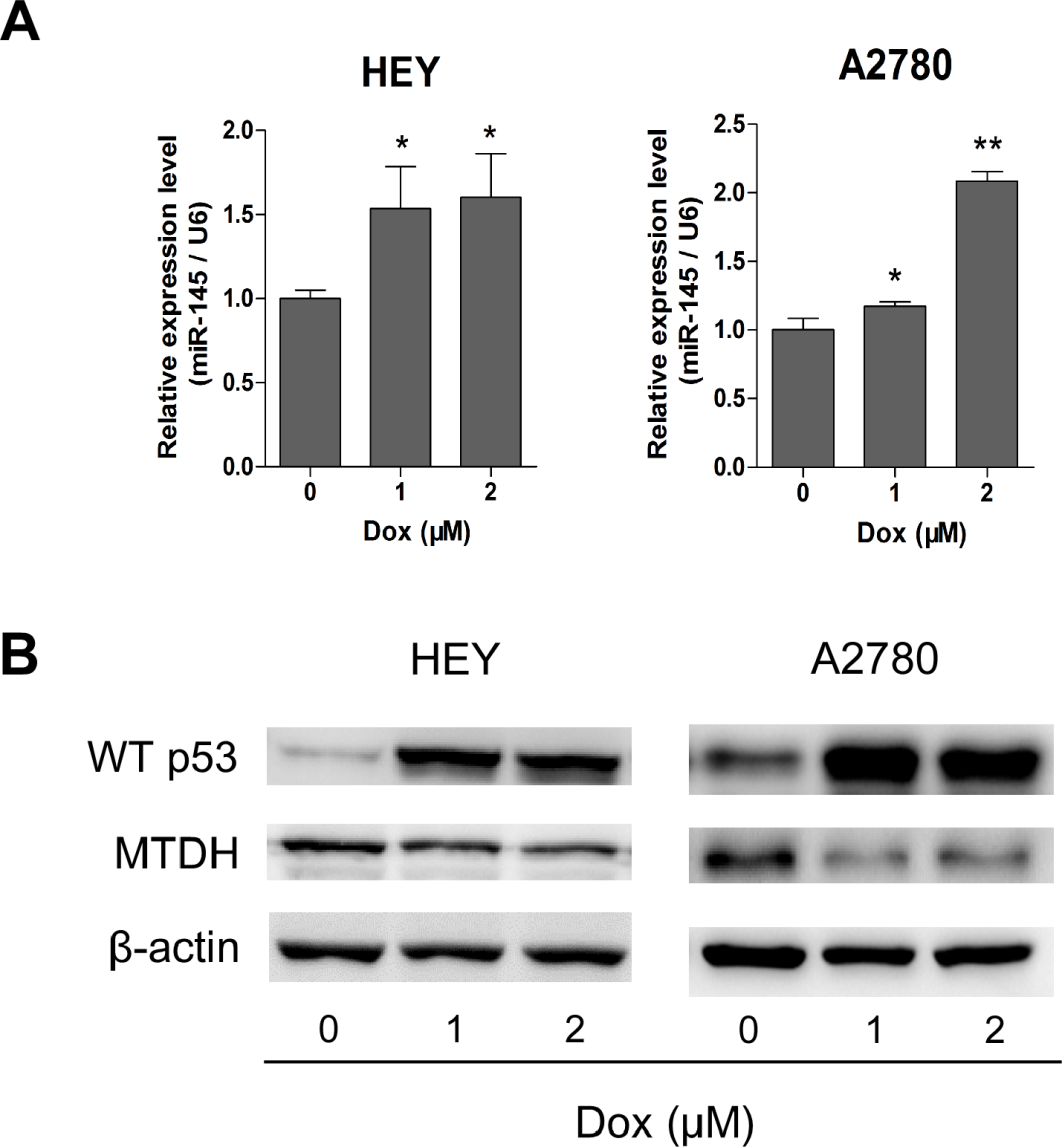
Wild type p53 represses MTDH through induction of miR-145 **(A)** Doxorubicin (Dox) was applied to induce p53 up-regulation, and qPCR assay shows that miR-145 can be significantly overexpressed after Dox utilization. **(B)** Western blot analysis of p53 and MTDH expression after treatment of Dox in HEY and A2780 cells. *P<0.05, **P<0.01.

Based on studies that p53 up-regulates the expression of miR-145[17] and our own finding that MTDH was directly targeted by miR-145, we proposed that p53 may repress MTDH expression through miR-145 in HGSOC. To test this, we measured the expression of p53, miR-145 and MTDH in ovarian cancer cells (with wild type p53 expression) treated with doxorubicin, a p53 activator[18]. As shown in Figure 7A and 7B, miR-145 expression was increased and MTDH expression was decreased when p53 was induced by doxorubicin. These findings suggest that in cells with normal p53, MTDH expression can be indirectly repressed by wild type p53.

### High MTDH expression correlates with poor prognosis of HGSOC patients

**Figure 8:**
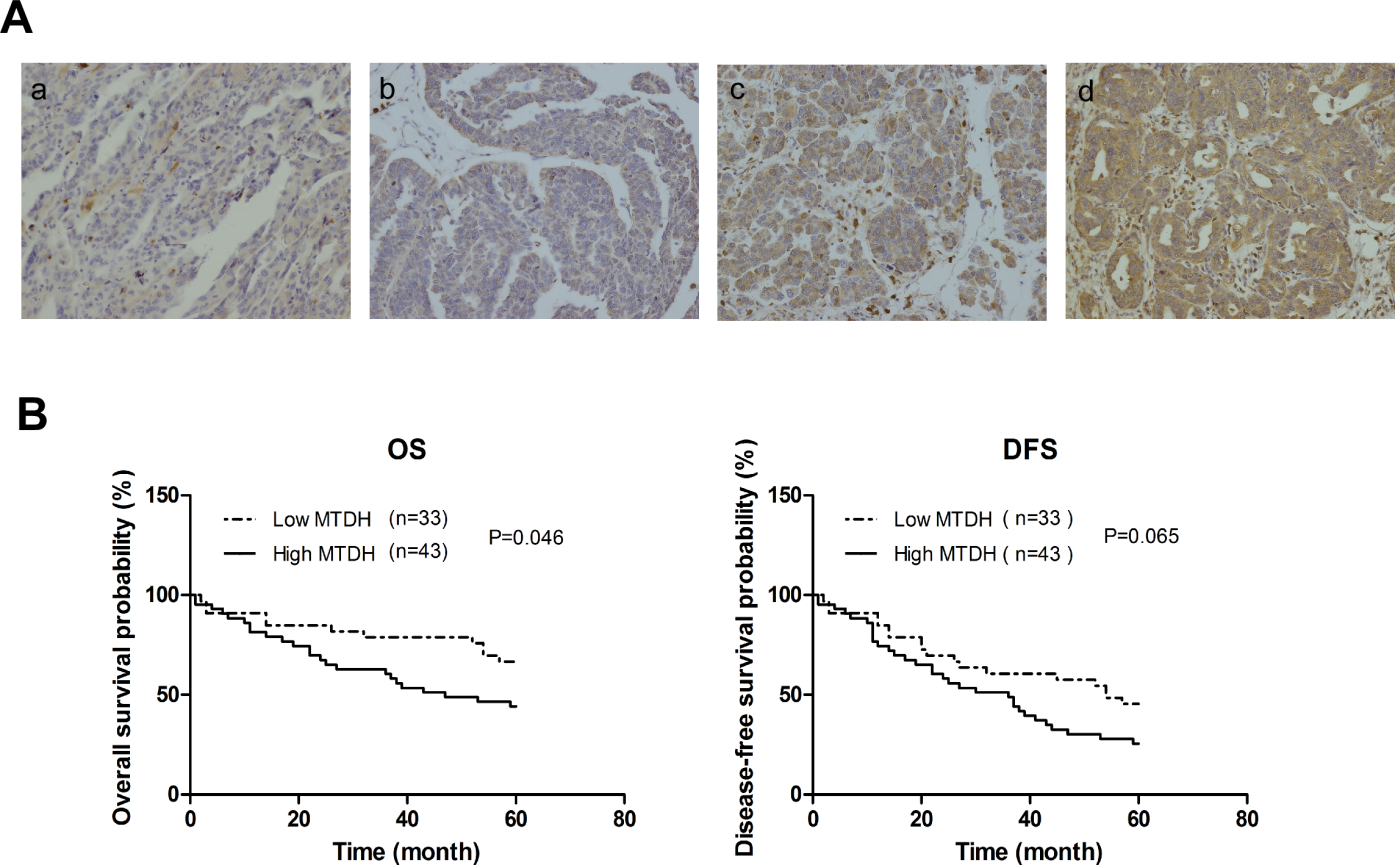
Expression level of MTDH in HGSOC samples correlates with prognosis of patients **(A)** Representative images of immunohistochemical analysis of MTDH expression in HGSOC patients samples. (a: score=0, b: score=1, c: score=2, and d: score=3). **(B)** Kaplan–Meier plots of overall survival, and disease-free survival for HGSOC patients dichotomized on the basis of high (score 2 and 3) versus low (score 0 and 1) MTDH immunohistochemical expression level.

To determine the clinical significance of MTDH in HGSOC, we examined the correlation between MTDH expression in HGSOC tumor samples (Figure 8A) and the clinical outcome (n=76). The patients were dichotomized into two categories according to their immunoreactivity for MTDH. Then overall survival (OS) and disease-free survival (DFS) rates were estimated by Kaplan-Meier survival curves. As can be seen from Figure 8B, patients with high expression of MTDH had shorter overall survival and disease-free survival period than those with low expression of MTDH. However, only overall survival reached statistical significance(P=0.046). These findings suggest that MTDH overexpression is a poor prognostic marker in HGSOC patients.

## DISCUSSION

miRNAs were deregulated in ovarian carcinoma[7, 19] and some altered miRNAs were associated with chemoresistance and survival[20, 21]. Here, we demonstrated that miR-145 was frequently down-regulated in HGSOC. We showed that MTDH was directly targeted by miR-145. Furthermore, p53 repressed MTDH through induction of miR-145. Importantly, we found that high MTDH expression level correlated with poor prognosis of HGSOC patients.

miR-145 was down-regulated in many malignancies including ovarian cancer[19]. However, the role of miR-145 in HGSOC remains to be determined. p53 directly and indirectly regulates the expression of a large number of miRNAs including miR-34, miR-200 families as well as miR-145[15]. p53 could also regulate miRNA processing and maturation as for miR-16-1, miR-143 and miR-145[22]. Transcriptional regulation of miR-145 seems to be tissue specific. In mouse, miR-145 and miR-143 are co-transcribed by Srf, myocardin and Nkx2.5 in multipotent cardiac progenitors before becoming localized to smooth muscle cells[23]. In human vascular smooth muscle cell, both TGF-β and BMP4 activate transcription of the miR-143/145 gene cluster[24]. In prostate cancer, miR-145 is regulated by DNA methylation and p53 gene mutation [17]. Since TP53 mutations occurred in almost all HGSOC (96%), down-regulation of miR-145 in HGSOC might be related to p53 mutation. In this study, p53 activation by doxorubicin caused an up-regulation of miR-145 and a down-regulation of MTDH in ovarian cancer cells (Figure 7A and 7B), which is consistent with the idea that down-regulation of miR-145 may be caused by p53 mutation in HGSOC.

In this study, we identified MTDH as a direct and functional target of miR-145 in HGSOC. Many studies demonstrated that MTDH might play important roles in the pathogenesis, progression, apoptosis regulation, angiogenesis, invasion, metastasis, and overall patient survival in diverse human cancers[25]. A recent study showed that MTDH played a critical role in mammary tumorigenesis by regulating oncogene-induced expansion and activities of tumor-initiating cells (TICs) through interacting with SND1[13]. Multiple mechanisms have been identified to contribute to MTDH overexpression in cancers. Genomic amplification of MTDH has been detected in breast and prostate cancers [16, 26]. Oncogenic Ha-ras could induce MTDH expression through the PI3K-Akt signaling pathway[27]. Other studies showed MTDH was regulated by miRNAs including miR-26a, miR-375, miR-136, and, miR30a[28–30]. Here we identified miR-145 as a new miRNA targeting MTDH.

In summary, we showed that the p53/miR-145 axis regulated MTDH expression in HGSOC. We demonstrate that MTDH, which is overexpressed in HGSOC, is directly targeted by miR-145. Importantly, high MTDH expression correlates with poor prognosis of HGSOC patients. Our current study provides a new link between p53, miR-145 and MTDH in the regulation of tumor growth and metastasis in HGSOC. Future studies are needed to assess the therapeutic effect of restoration of miR-145. MTDH can become a powerful prognosis marker for HGSOC.

## MATERIALS AND METHODS

### Patients and tissue samples

High-grade serous ovarian carcinoma (HGSOC) and fallopian tube tissues were collected in Qilu Hospital from April 2008 to July 2012. The HGSOC specimens were obtained from primary ovarian cancer patients receiving no surgery or chemotherapy previously. Fallopian tube tissues were from patients who received a total hysterectomy and bilateral salpingo-oophorectomy for uterine diseases or for benign neoplastic adnexal pathologic changes. All the fresh samples were obtained at surgery, immersed in RNAlater ( Ambion) and stored at −80°C. Ethics Committee of Shandong University approved the study and all participants gave written informed consent. The clinicopathologic characteristics were shown in Supplementary Table S2.

### Microarray analysis

Five pairs of collected tissues (fimbria and HGSOC) underwent microarray analysis. Total RNA was harvested using Trizol reagent (Invitrogen) and miRNeasy mini kit (QIAGEN) according to manufacturer's instructions. After having passed RNA quantity measurement using the NanoDrop 1000, the samples were labeled using the miRCURY™ Hy3™/Hy5™ Power labeling kit and hybridized on the miRCURY™ LNA Array (v.18.0). Following the washing steps the slides were scanned using the Axon GenePix 4000B microarray scanner. Scanned images were then imported into GenePix Pro 6.0 software (Axon) for grid alignment and data extraction. Replicated miRNAs were averaged and miRNAs that intensities >=30 in all samples were chosen for calculating normalization factor. Expressed data were normalized using the Median normalization. After normalization, significant differentially expressed miRNAs were identified through Volcano Plot filtering. Finally, hierarchical clustering was performed to show distinguishable miRNA expression profiling among samples. The microarray data generated in this study have been deposited in NCBI GEO database under the accession number GSE61485.

### Cell lines and cell culture

HO8910, HO8910PM, OVCAR3 and HEK293T cells were purchased from China Type Culture Collection (Shanghai,China). FTE187 (immortalized normal human fallopian tube epithelial cell line) cell line was from Jinsong Liu's lab as described[31]. SKOV3, HEY, A2780 and Phoenix amphotropic cells (retroviral packaging cells) were from Jian-Jun Wei's lab as previously described[8]. FTE187 cell line was maintained in cell culture medium consisting of 1:1 Medium199 (Sigma-Aldrich) and MCDB105 medium (Sigma-Aldrich) with 10% heat-inactivated fetal bovine serum (FBS, Gibco) and 10 ng/ ml epidermal growth factor (Sigma-Aldrich). SKOV3, HO8910, OVCAR3 and HO8910PM cells were cultured in RPMI 1640 medium (Gibco). HEK293T, HEY and A2780 cells were cultured in Dulbecco's modified Eagle's medium (DMEM) (Gibco). All media contained 10% FBS, 100 μg/ ml penicillin, and 100 μg/ml streptomycin. All cells were cultured at 37°C with 5% CO2 in a humidified incubator (Thermo Fisher Scientific).

### Plasmid construction and transfection

To generate a miR-145 expression vector, a 487 bp fragment containing pre-miR-145 was cloned into pGIPZ lentiviral vector (Open Biosystems). Lentivirus expressing miR-145 or negative control (NC) was produced in HEK293T cells packaged by pMD2G and psPAX2. For stable infection, 1×10^5^ cells were plated in six-well plates along with 2 mL of medium without antibiotics. After overnight incubation, the medium was removed and replaced with 1 mL per well of Opti-MEM Reduced-Serum Medium (Gibco) containing 8 μg/mL polybrene. Next, 50 μL of concentrated lentiviral particles were added to each well. Twenty-four hours later, fresh medium containing 2 μg/mL puromycin (Sigma-Aldrich) was added to each well. Fresh medium containing puromycin was replaced every 3 to 4 days. Single colonies were obtained after 3 weeks of puromycin selection. For MTDH overexpression, the retroviral expression vector pBabe-hygro was used. pBabe-hygro-MTDH overexpression plasmid was a gift from Dr. Guohong Hu. Viruses were generated by Phoenix amphotropic cells and used to infect target cells as above except that the infected cells were selected with 200ug/ml hygromycin (Roche). Primers for plasmid construction are shown in Supplementary Table S3.

### Transient transfection

Mimics (Gene Pharma) and inhibitor (Invitrogen) of miR-145 and the corresponding negative control were used to achieve the transient overexpression or knockdown of miR-145. Cells were transfected with lipofectamine 2000 (Invitrogen) according to the manufacturer's protocol. Transfection was performed at 50nM for mimics and 1-100nM for inhibitor.

### RNA isolation and Quantitative real-time PCR

Total RNA was extracted from cultured cells or fresh tissues with Trizol reagents (Ambion) according to the manufacturer's protocol. The cDNA of miRNA was synthesized with One Step PrimeScript miRNA cDNA Synthesis Kit (Takara). Quantitative real-time PCR (qPCR) was performed with the SYBR green Premix Ex Taq II (Takara) with StepOne Plus Real-Time PCR System (Applied Biosystems). The expression of U6 was used as the endogenous control for detection of miRNA expression level. Primer information used in the study can also be found in Supplementary Table S3.

### Western blot analysis

Cells were lysed and protein concentration was determined using the BCA Assay Kit (Thermo Scientific). Protein samples were separated by SDS-PAGE and electrotransferred onto PVDF membrane (Millipore). After blocking with 5% non-fat milk, the membrane was incubated overnight at 4°C with the primary antibody and then with horseradish peroxydase-coupled secondary antibody. Signal was detected with enhanced chemiluminescence (ECL) (PerkinElmer) by ImageQuant LAS 4000 (GE Healthcare Life Sciences). Antibodies used include anti-MTDH (Invitrogen), anti-P53 (DAKO), anti-β-actin (Sigma-Aldrich). Other antibodies used in this study were from Cell signaling Technology.

### Migration and invasion assays

Migration and invasion assays were performed using transwell system (24-well, 8μm pore size; BD falcon) according to manuals. Briefly 5-10 ×10^4^ cells were seeded into the upper chambers of the transwell with certain medium containing no FBS, and lower chambers were filled with culture media containing 20% FBS as a chemo-attractant. The chambers were incubated at 37°C for 4–48 hours depending on the cell lines type. Successfully translocated cells were fixed by methanol for 15 minutes, stained with 0.2% crystal violet for 30 minutes, and counted under a light microscope.

### MTT assay

The proliferation ability of cells was measured using 3-[4, 5-dimethylthiazol-2-yl]-2, 5-diphenyltetrazolium bromide (MTT) assay. Cells were seeded into 96-well plates (1.5-3×10^3^cells/well) for continual 1–5 days. At specified time points, 20μl of MTT (Sigma-Aldrich) solution (0.5 mg/ml) was added to each well, and the cells were incubated for additional 4 hours at 37°C. Then the supernatants were carefully removed and 100μl of dimethyl sulfoxide (DMSO, Sigma-Aldrich) was added to each well. The absorbance values were evaluated at 490nm with a Microplate Reader (Thermo Fisher Scientific).

### Clonogenic assay

Clonogenic assay was performed as described previously[32]. Briefly, single-cell suspensions were generated for each cell line and specified numbers of cells were seeded into six-well tissue culture plates. Then cells were cultured for 10 days. Colonies were stained with crystal violet. Colonies of greater than 50 cells were counted. The data presented is the mean ± standard error (SE) and represents three independent experiments.

### Luciferase reporter assay

The 3′UTR of MTDH containing two putative miR-145 binding sites (580bp) was amplified and cloned into pmirGLO vector (Promega) using the SacI and XbaI sites to generate the wild type construct [1]. For mutant plasmid (MT), overlap extension PCR assay [33] was used as described previously [34]. Cells were cultured in 96-well plates and transfected with 100ng of WT or MT MTDH 3′UTR constructs by lipofectamine 2000 assay. Twenty-four hours after transfection, luciferase activity was measured using the Dual-Glo Luciferase Assay System (Promega). Renilla luciferase activity was normalized to corresponding firefly luciferase activity and plotted as a percentage.

### *In vivo* nude mice tumorigenesis and metastasis assay

For xenograft experiment, 5× 10^6^ cells in 120μl mixed solution (PBS: Matrigel (BD falcon) =5:1) were injected subcutaneously into 4–5 weeks-old BALB/c nu/nu female mice. Tumor growth was monitored by measuring tumor diameters. Tumor volume was calculated according to the formula TV(cm3)=a×b2×π/6, where a is the longest diameter, and b is the shortest diameter. Mouse was euthanized when a tumor reached 2 cm in diameter. To produce experimental lung metastasis, 2 × 10^6^ cells in 100μl PBS were injected into the lateral tail veins of 4–5 weeks-old BALB/c nu/nu female mice. 6 weeks later, mice were sacrificed under anesthesia. The lungs were collected and fixed in 10% formalin. For tissue morphology evaluation, hematoxylin and eosin (HE) staining was performed on sections from embedded samples. All animal experiments were performed with the approval of Shandong University Animal Care and Use Committee.

### Immunohistochemistry

Formalin-fixed and paraffin-embedded tissues were sectioned at 4 μm. Tissue slides were deparaffinized in xylene and rehydrated in a graded series of ethanol. Antigen retrieval was performed by heat-induced epitope retrieval. All immunohistochemical staining procedures were performed on a Ventana Nexus automated system. After blocking in 1.5% normal goat serum for 30 minutes at room temperature, slides were then incubated overnight at 4°C with primary antibodies of MTDH (Invitrogen ) in a humid chamber. Staining was detected with I-View 3,3′-diaminobenzidine (DAB) detection system. MTDH staining results were scored based on staining intensity (0 = negative, 1 = weak, 2 = moderate, 3 = strong).

### Statistics analysis

The software SPSS V20.0 was used for statistical analysis. Student's t-test and one-way ANOVA analysis were used to determine significance. P < 0.05 was considered statistically significant.

## SUPPLEMENTARY TABLES




