# Supplementary material for: miR-145 inhibits tumor growth and metastasis by targeting metadherin in high-grade serous ovarian carcinoma
Source: Oncotarget. 2014 Oct 23;5(21):10816–29. doi: 10.18632/oncotarget.2522 (PMC4279412; doi:10.18632/oncotarget.2522)
Supplement: Supplementary file 1 [file oncotarget-05-10816-s001.pdf]

## SUPPLEMENTARY TABLES

### Supplementry Table S1. HGSOC vs Fimbria 2.0 fold up regulated miRNAs

## Supplementry Table S2. Main clinicopathological characteristics of patients enrolled in this study

| | Serous ovarian carcinoma | Fimbria |
|---|---|---|
| Age (yrs)<br>Mean±SD | 58.8±9.5 | 55.4±10.4 |
| Median | 59.5 | 55 |
| Range | 41–76 | 34–78 |
| Case number | Total 124 | Total 19 |
| | Fresh tissues 48 | Benign changes of endometrium 4 |
| | Follow-up cohort 76 | leiomyoma 8 |
| | | Benign tumors of ovary 5 |
| | | Endometrium ectopia 2 |

**Supplementry Table S3. Primers used in this study**

| Method | Primer Name | Primer sequence(5′—3′) |
|---|---|---|
| Construction | GIPZ-puro- miR-145 (F) | CCTAGCGTTAA**C** CGCCAGAGGGTTTCCGGTACT |
| Construction | GIPZ-puro- miR-145 (R) | GATCCGCTCGAG ACGGCAGTGCTGAAGTTCCCA |
| Construction | MTDH 3′UTR-WT (F) | GCATATGAGCTCGCTCCCTAATCCAGCCTGTC |
| Construction | MTDH 3′UTR-WT (R) | GCTCTAGACTGACAAAGTCCCTCCCCAC |
| Construction | MTDH 3′UTR-MT 1(F) | GTTTAGGGGTTTCTCCAGcagaacATCCTACCTATCTGTAC |
| Construction | MTDH 3′UTR-MT 1 (R) | GTACAGATAGGTAGGATgttctgCTGGAGAAACCCCTAAAC |
| Construction | MTDH 3′UTR-MT 2 (F) | GTAAACTCACTGCACAgcagaacATTACTTTCCAAAAG |
| Construction | MTDH 3′UTR-MT 2 (R) | CTTTTGGAAAGTAATgttctgcTGTGCAGTGAGTTTAC |
| qPCR | miR-145(F) | GTCCAGTTTTCCCAGGAATCCCT |
| qPCR | MTDH (F) | AAATAGCCAGCCTATCAAGACTC |
| qPCR | MTDH (R) | TTCAGACTTGGTCTGTGAAGGAG |
| qPCR | U6(F) | AACGCTTCACGAATTTGCGT |
| qPCR | GAPDH (F) | TGCACCACCAACTGCTTAGC |
| qPCR | GAPDH (R) | GGCATGGACTGTGGTCATGAG |
